# *In Vitro* Evaluations and* In Vivo* Toxicity and Efficacy Studies of MFM501 against MRSA

**DOI:** 10.1155/2017/8032865

**Published:** 2017-04-27

**Authors:** Saiful Azmi Johari, Mastura Mohtar, Sharifah Aminah Syed Mohamad, Mohd Fazli Mohammat, Rohana Sahdan, Azman Mohamed, Mohamad Jemain Mohamad Ridhwan

**Affiliations:** ^1^Antimicrobial Laboratory, Anti-Infective Branch, Bioactivity Programme, Natural Products Division, Forest Research Institute Malaysia (FRIM), 52109 Kepong, Selangor, Malaysia; ^2^Faculty of Applied Sciences, Universiti Teknologi MARA (UiTM), 40450 Shah Alam, Selangor, Malaysia; ^3^Organic Synthesis Laboratory, Institute of Science, Universiti Teknologi MARA (UiTM), 40450 Shah Alam, Selangor, Malaysia; ^4^Biotherapeutic Branch, Bioactivity Programme, Natural Products Division, Forest Research Institute Malaysia (FRIM), 52109 Kepong, Selangor, Malaysia; ^5^Pharmacy Programme, Sultan Azlan Shah Allied Health Sciences College, 31250 Tanjung Rambutan, Perak, Malaysia

## Abstract

Previously we have discovered a synthetically derived pyrrolidone alkaloid, MFM501, exhibiting good inhibitory activity against 53 MRSA and MSSA isolates with low cytotoxicity against three normal cell-lines with IC_50_ values at >625 *µ*g/ml. Time-kill assay, scanning electron microscopy (SEM) analysis,* in vivo* oral acute toxicity test, and mice peritonitis model were carried out in this study. In the time-kill study, MFM501 showed a less than 3 log_10_ decrease in bacterial colony concentration value (CFU/ml) which represented a bacteriostatic action while displaying a time-dependent inhibitory mechanism. Following that, SEM analysis suggested that MFM501 may exert its inhibitory activity via cytoplasmic membrane disruption. Moreover, MFM501 showed no toxicity effect on treated mice at an estimated median acute lethal dose (LD_50_) value of more than 300 mg/kg and less than 2000 mg/kg. For the efficacy test, a mean effective dose (ED_50_) of 87.16 mg/kg was obtained via a single dose oral administration. Our data demonstrated that MFM501 has the potential to be developed further as a new, safe, and effective oral-delivered antibacterial agent against MRSA isolates.

## 1. Introduction

Methicillin-resistant* Staphylococcus aureus* (MRSA) are nosocomial-related, Gram-positive bacteria that have been known to display multidrug-resistance (MDR) properties towards a wide range of structurally unrelated antibiotics and antimicrobial agents. Currently, only a handful of antibiotics could inhibit this dangerous pathogen. Previously, we have discovered a synthetically derived pyrrolidone alkaloid, MFM501, exhibiting good inhibitory activity with MIC values between 15.6 and 31.3 *µ*g/ml against 38 MDR MRSA and 13 methicillin-sensitive* S. aureus* (MSSA) isolates with low cytotoxicity against three normal cell-lines (WRL-68, Vero, and 3T3) with IC_50_ values at >625 *µ*g/ml [1].

Nevertheless, MIC measurement could not determine the rate at which a candidate compound kills bacteria [2]. Due to that, time-kill curves were plotted to specifically determine the kinetics of time-dependent or concentration-dependent bacterial killing of MFM501 against a representative MRSA isolate. In addition, although unconventional, time-kill curves could help determine the bacteriostatic or bactericidal action of the potential derivatives [3]. Subsequently, a scanning electron microscopy (SEM) analysis was chosen to evaluate the antibacterial effect from MFM501 on the cell structure of a selected MRSA isolate since earlier study has shown that the pyrrolidone ring plays an important role for the strong binding affinity to the penicillin-binding-protein 2a (PBP2a) site which could prevent cell wall synthesis in Staphylococci species as exemplified in ceftobiprole, a commercially available fifth-generation cephalosporin antibiotic which has a pyrrolidone ring on its chemical structure [4, 5].

On the other hand, although* in vitro* cytotoxicity assay has several advantages such as less experimental time and monetary consumption, it could not disclose the harmful and systemic effect of a compound against certain organs as in* in vivo* toxicity studies [6]. Most importantly, there were no definitive and precise procedures for* in vitro *toxicity tests given by regulatory authorities as compared to* in vivo* toxicity assays that were regulated by the Organization for Economic Cooperation and Development (OECD) [6]. In view of that, the fixed dose procedure for acute oral toxicity number 420 from the OECD Guidelines for the Testing of Chemicals was selected since it was the recommended initial animal toxicity study that could provide critical data on the relative toxicity likely to arise from a single or brief exposure to MFM501 [7]. An estimated range of mean lethal dose (LD_50_), lowest fixed dose causing evident toxicity by the tested compounds, will be determined [8].

To evaluate the efficacy of MFM501 in an animal model, the systemic infection assay was chosen due to its simple end points (death or survival) and availability of results within 48 h [9]. More importantly, preclinical assay in antibacterial development is a must before moving to tests in larger animals or human because the mice immune system is very similar to humans [10]. In this study, further evaluations on MFM501 against selected MRSA isolates were carried out to determine its microbiological, safety, and efficacy profiles.

## 2. Material and Methods

### 2.1. Preparation of MFM501

As described in previous study, MFM501 was synthesized in the Organic Synthesis Laboratory, Institute of Science (IOS), UiTM, Shah Alam, and identified using NMR and FTIR methods [1].

### 2.2. Bacterial Isolates and Growth Conditions

The MRSA ATCC 33591 reference strain was employed in the time-kill assay as well as the infectious agent in the* in vivo* systemic infection studies. For SEM analysis, MRSA strain ATCC BAA-1688 was utilized. Isolates were maintained in the Antimicrobial Laboratory, FRIM, on Protect Bacterial Preservers (Technical Service Consultants Limited, Heywood, Lancashire, England) at −20°C. Prior to use, isolates were subcultured overnight at 37°C in Mueller-Hinton broth (MHB) and adjusted to obtain turbidity comparable to that of McFarland standards accordingly using a cell density meter (Biochrom WPA CO8000, Cambridge, UK) at 600 nm.

### 2.3. Time-Kill Assay

MFM501 was evaluated for inhibitory effect at (1/2)x, 1x, and 2x MIC value over 24 h and the growth profile curve was plotted. In this experiment, a more simplified, faster, and cost effective track-dilution method was employed as described previously [11, 12]. During a MIC assay, a 10 *µ*l sample from each respective well representing a (1/2)x, 1x, and 2x MIC value after 24 h was spotted on an imaginary line on one end of a preprepared conventional MHA plate. After depositing the last sample, the plate was tilted at a 45° angle so that the spots were allowed to gravitate across the MHA plate. Subsequently, the tracks were left to dry for 1 min and incubated in the usual inverted position at 37°C for 24 h. Lanes with the highest number of readily distinguishable and separate colonies were used to obtain the final count. All samples were evaluated in triplicate.

### 2.4. Scanning Electron Microscopy (SEM) Analysis

The effects of the active compound/s on MRSA cell structure at (1/2)x MIC and 1x MIC values were estimated at the Microscopy Unit, Institute of Bioscience, Universiti Putra Malaysia (UPM). Firstly, a 20 ml of 24 h MRSA culture was harvested and centrifuged and the supernatant was discarded. After that, 2.5% glutaraldehyde was added to the pellet, mixed thoroughly, and incubated at 4°C for 4–6 h. Secondly, the pellet was washed using 0.1 M sodium cacodylate for 3x of 10 min of incubation at room temperature. Next, postfixation was done by adding 1% osmium tetraoxide onto the pellet, with thorough mixing and incubation at 4°C for 2 h. Similarly, the pellet was washed again using 0.1 M sodium cacodylate. Dehydration step was carried out by subjecting the washed pellet to a series of acetone with serial dilution of 35%, 50%, 75%, and 95% for 10 min each, and 100% acetone for 15 min (3x) at room temperature. The final cell suspension was pipetted onto an aluminium foil and dried in a Critical Point Dryer (Baltec-030 CPD). The dried specimens were then fixed on the stub and coated with gold under vacuum by a sputter unit (Baltec SCD 005) before SEM viewing using the JEOL-JSM 6400 Scanning Electron Microscope at 15 kV acceleration voltages was carried out.

### 2.5. Animals and Living Conditions

Seven-to-eight-week-old healthy imprinting control region (ICR) mice with body weight of 20–30 g were employed in the experiment. Animals were housed in polypropylene cages with stainless steel grills while for the infection study, mice were housed in polypropylene individually ventilated cages (IVC) housing system to limit cross-infection between the cages and animal handlers. Mice were acclimated for a minimum period of five days in the controlled environment (temperature: 22 ± 3°C) with relative humidity between 30% and 70% and 12 h light : 12 h dark cycle. Ad libitum water and standard rodent pellets were supplied to the animals while corn cobs were used as bedding material. All mice experiments in this study were approved by both UiTM's Committee in Animal Research and Ethics (CARE; Ref. number 27/2013) and FRIM's Animal Care and Use Committee (ACUC; Ref. number IACUC-FRIM/1(2013)/01).

### 2.6. Acute Toxicity Test

The fixed dose procedure for oral acute toxicity was employed as recommended by the OECD [13]. For the sighting study, one randomly selected female mouse was orally given a fixed recommended dose of 2000 mg/kg body weight of MFM501 following a period of fasting. A 5% Tween 80 solution was used as the dosing vehicle in the study. Observations were carried out at 30 min, 2 h, 4 h, 6 h, and 24 h. If the mouse did not survive the initial dosage, a lower recommended dosage of 300 mg/kg would be applied. The main study was carried out using identical concentration, of either 2000 mg/kg or 300 mg/kg, which the tested mouse has survived. Observations were made at 30 min, 4 h, and daily observations up to 14 days/dose intended for any signs of toxicity such as changes of skin and fur, abnormal behaviors, and death. The LD_50_ value was estimated based on the Globally Harmonized System of Classification and Labeling of Chemicals (GHS) which dictates the lethal dose of a chemical, given all at once, which causes the death of 50% of the test animal [14].

### 2.7. Blood and Organ Analysis

At the end of the experiment, blood was collected from untreated (control) and treated mice through cardiac puncture procedure in EDTA-coated tubes for both hematology and biochemistry analysis on the 14th day following a 12 h fast. The hematological analysis includes the white blood cell (WBC) and red blood cell (RBC) counts; lymphocyte (Lymph), monocyte (Mon), and granulocyte (Gran); the hemoglobin (HGB) and hematocrit (HCT) levels, mean corpuscular volume (MCV), mean cell hemoglobin (MCH), mean cell hemoglobin concentration (MCHC), and red blood cell distribution width (RDW); number of platelets (PLT), mean platelet volume (MPV), platelet distribution width (PWD), and plateletcrit (PCT) were determined using a Mindray BC*-*2800Vet Auto Hematology Analyzer (Mindray Corporation, Shenzhen, China).

Consequently, the same whole-blood containing tubes were centrifuged at 4000 rpm (10 min at RT) and the supernatant was collected and introduced into new tubes for the subsequent biochemical analysis for the levels of total protein (TP), albumin (ALB), alanine phosphatase (ALP), alanine transaminase (ALT), aspartate transaminase (AST), uric acid (UA), high density lipoprotein (HDL), triglycerides (TG), and glucose (GLU). These parameters were measured using reagent kits and a Roche Cobas C111 Clinical Chemistry Analyzer (Indianapolis, Indiana, USA). Lastly, organs (heart, thymus, lung, liver, kidneys, spleen, intestine, ovaries, and brain) of treated and untreated mice were harvested. The mean relative weight of each organ to its respective body weight and macroscopic evaluation of each organ were compared between treated and untreated mice.

### 2.8. Statistical Analysis

The data were expressed as the mean ± standard deviation (SD) and were analyzed using the GraphPad Prism version 5 (GraphPad, San Diego, CA, USA) to account for the effects of MFM501 on the weight (of the mice and organs of the mice) and hematological and biochemical findings. Results with *p* < 0.05 will be considered as statistically significant.

### 2.9. Mouse Systemic Infection Assay

This study was performed as described previously with minor modifications [15, 16]. A group of six ICR mice was given a MRSA adjuvant via intraperitoneal (i.p.) route. A MRSA adjuvant consisted of a standardized 1.2 × 10^9^ CFU/ml MRSA culture suspended in equal volume of 5% mucin. MFM501 was prepared in 5% Tween 80 and dissolved into four serial concentrations between 15.6 mg/kg and 125 mg/kg. Subsequently, the test compounds were administered in single dose via oral route (p.o.) 1 h after i.p. infection. The number of mice that survived was observed over seven days. The total number of survivors at each dose was used to calculate the mean effective dose (ED_50_) value. The ED_50_ determinations were performed by GraphPad analysis within each test.

## 3. Results

**Figure 1:**
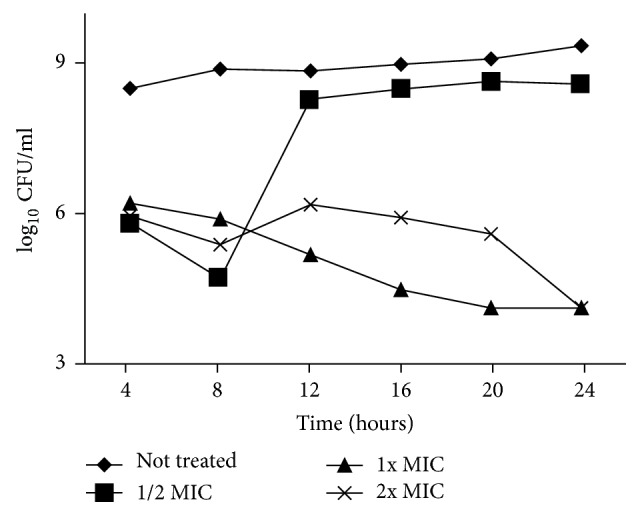
Time-kill curves for MFM501 against MRSA ATCC 33591.

The result of the time-kill kinetics for MFM501 was depicted in Figure 1. The killing rate of MFM501 at 1x MIC was <3 log_10_ CFU/ml reduction from the initial CFU count (6.431 log_10_ CFU/ml) at either 20 h or 24 h (4.114 log_10_ CFU/ml). Similar reduction rate of <3 log_10_ CFU/ml at 24 h was observed for MFM501 at 2x MIC concentration although a rebound effect was observed during 12 h, 16 h, and 20 h of the experiment. On the other hand, even though the initial inhibitory rate of MFM501 at (1/2)x MIC was lower than 1x and 2x MIC, the growth rate increased significantly after 8 h and steadily continued up to 24 h. Based on the plotted graph, a concentration-dependent killing mechanism was observed for MFM501 since, at 20 h, different log_10_ CFU/ml values were observed for either 1x MIC or 2x MIC concentrations.

**Figure 2:**
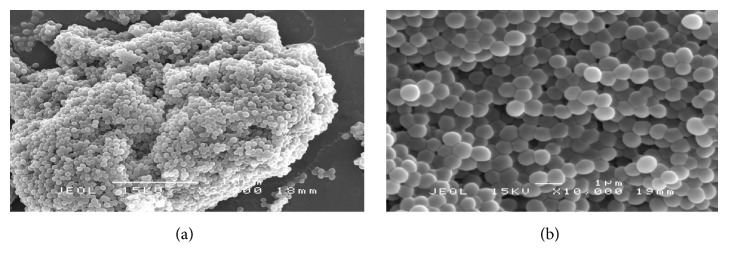
(a) Untreated MRSA cells after 24 h of incubation (magnification: 3,000x). (b) Untreated MRSA cells after 24 h of incubation (magnification: 10,000x).

**Figure 3:**
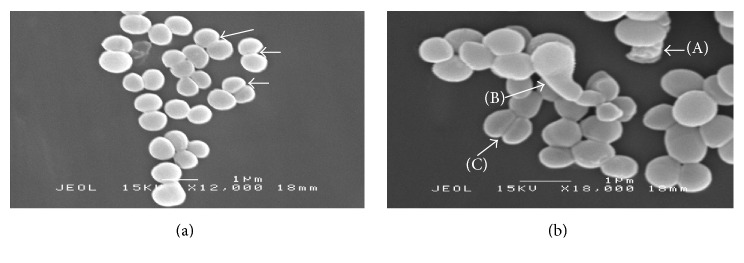
(a) MRSA cells treated with (1/2)x MIC value of MFM501 after 24 h of incubation (magnification: 12,000x). Arrows showing disruption of cell wall division. (b) MRSA cells treated with (1/2)x MIC value of MFM501 after 24 h of incubation (magnification: 18,000x). Arrows showing (A) crinkling of cell wall and (B) elongated cell (C) disruption of cell wall division.

**Figure 4:**
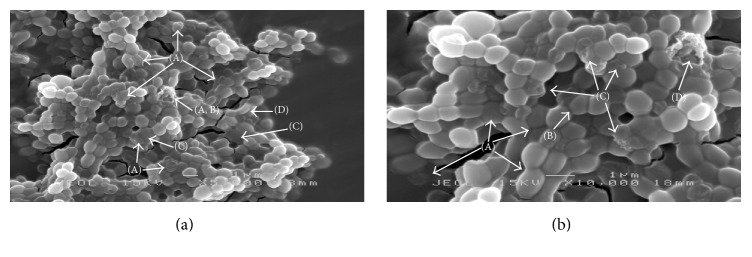
(a) MRSA cells treated with 1x MIC value of MFM501 after 24 h of incubation (magnification: 5,000x). Arrows showing (A) irregular and shrunken cells, (B) membrane disruption, (C) biofilm production, and (D) elongated cells. (b) MRSA cells treated with 1x MIC value of MFM501 after 24 h of incubation (magnification: 10,000x). Arrows showing (A) biofilm formation, (B) disruption of cell wall division, (C) distorted cell structures, and (D) cell lysis with membrane disruption.

In the SEM analysis, two concentrations of MIC values for MFM501 were employed to qualitatively observe the structural changes and differences between the treated and nontreated MRSA cells. The results for untreated MRSA bacterial cells were presented in Figure 2 while treated MRSA cells with MFM501 at (1/2)x MIC and 1x MIC values were presented in Figures 3 and 4, respectively. The untreated MRSA cells (ATCC BAA-1688) showed a normal, uniformed, and intact cell shape with an undamaged spherical structure with smooth cell surface in a grape-like cluster (Figures 2(a) and 2(b)). Conversely, at (1/2)x MIC value of MFM501, treated MRSA cells exhibited several cell wall division interruptions as clearly presented in Figure 3(a). Additionally, an elongated cell and crinkling of cell wall due to disruption of cell wall division were also detected in Figure 3(b). Interestingly, the treated cells at (1/2)x MIC value showed slightly enlarged cell diameters as compared to untreated cells.

At 1x MIC value, the majority of MFM501 treated cells appeared to be engulfed by the extracellular matrix of bacterial biofilm while irregular, shrunken, and elongated cells were also present as seen in Figure 4(a). Upon closer inspection (Figure 4(b)), some treated cells clearly displayed cell lysis with membrane disruption, distorted cell structures, and incomplete cell wall divisions. Nevertheless, the ubiquitous biofilm was detected in almost all of the treated MRSA cells. The slight increase in size of the many treated cells was also detected at 1x MIC value against MFM501. However, occurrence of the destroyed MRSA cells by MFM501 at (1/2)x MIC value was much less as compared to the observable and numerous damaged MRSA cells at 1x MIC value.

In the acute toxicity study, the single ICR female mouse died within 30 min from the initial fixed dose of MFM501 at 2000 mg/kg. Occurrences of tremors and piloerection were observed immediately on the mice after oral gavage was performed. Following that, a lower dose of 300 mg/kg was administered to another randomly selected female mouse. Fortunately, mouse given 300 mg/kg dose of MFM501 did not reveal any toxicity symptoms or mortality up to 24 h. Based on the results of the sighting study, a dosage of 300 mg/kg was chosen for the main study. In this main experiment, mice given MFM501 at 300 mg/kg did not show any adverse effects or clinical signs of toxicity during the 14 days of the experiment. Similarly, mice that received 5% Tween 80 only did not exhibit any toxicity symptoms for the entire two weeks of the experiment. On another note, MFM501 did not show significant difference in the mice body weight at days 3, 7, 10, and 14 when compared to the untreated control group as listed in Table 1. All mice were sacrificed at the end of the study duration via carbon dioxide inhalation.

Hematological values obtained from treated and untreated mice were shown in Table 2. There were no significant differences in the hematological values of mice blood cells between untreated and treated mice with MFM501 at 300 mg/kg. Similarly, there were no significant differences in the renal and lipid profiles of mice blood cells between untreated and treated mice with MFM501 at 300 mg/kg as exhibited in Table 3. However, there was a significant increase of the AST enzyme from liver profile of mice treated with MFM501 at 300 mg/kg as compared to untreated mice.

On the other hand, gross macroscopic evaluation and mean relative weight of various organs from mice treated with MFM501 did not demonstrate any abnormal changes as compared to untreated mice as listed in Table 4. These results showed that although biochemical analysis has detected elevated AST enzyme level in mice treated with MFM501 at 300 mg/kg, this irregularity was not observable in the gross macroscopic examination of treated organs and it did not affect the relative organ weight of treated mice. Moreover, no toxicity symptoms were observed on the treated group of mice during the 14 days' duration of the experiment.

In the systemic infection assay, untreated mice challenged with MRSA adjuvant and treated with MFM501 at 15.6, 31.3, 62.5, and 125 mg/kg showed significant moderate survival rates of 25, 37.5, and 62.5% as displayed in Table 5. Interestingly, both 15.6 and 31.3 mg/kg of dosage gave similar 25% protection on infected mice. Nevertheless, MFM501 exhibited a dose-dependent protection trend in this study. However, MRSA-infected mice treated with 25 mg/kg linezolid showed 100% mice survival. Similarly, no mortality was observed in nontreated mice and mice given only MHB and 5% mucin. As expected, 100% mortality rate was detected on mice challenged with MRSA adjuvant and no treatment with MFM501 or linezolid.

Next, the mean effective dose (ED_50_) for MFM501 in MRSA-challenged ICR female mice was calculated using GraphPad analysis software. Based on the four serial concentrations of MFM501, the ED_50_ value was calculated at 87.16 mg/kg. As shown in Table 5, linezolid exhibited better protective ability with 100% survival rate of infected mice at 25 mg/kg dosage as compared to MFM501 that cured 62.5% of the infected mice population at 125 mg/kg, a threefold dosage increment as compared to linezolid. These results showed that linezolid was still the superior choice of antibiotic against MRSA infections. On the other hand, both active compounds could be an alternative option for oral administration to combat MRSA/MSSA infections.

## 4. Discussion

Previous studies have suggested that compounds which exhibited <3 log_10_ CFU/ml reduction from initial CFU count at 24 h indicated a bacteriostatic effect while compounds that displayed ≥3 log_10_ CFU/ml reduction from initial CFU count at 24 h indicated a bactericidal action against the tested microbe/s [17, 18]. Results of this time-kill study corroborated with previous MBC/MIC ratio values [1] that suggested MFM501 has a bacteriostatic mechanism of action against MRSA and MSSA isolates.

On the other hand, the rebound effect observed in this study was a common phenomenon in bacterial killing rate studies involving antibacterial agents [19, 20]. This occurrence may due to two distinct bacterial subpopulations with different susceptibility against tested antimicrobial agents in which the selective growth of resistant subpopulation takes over the preferential killing of the susceptible subpopulation at a specified time of interaction [19].

The various irregular, distorted, and shrunken shapes of the treated MRSA cells detected in this study may be attributed to the loss of cellular contents due to bacterial lysis and/or cytoplasmic membrane disruptions [21, 22]. Similar irregular structures of MRSA bacterial cells were observed when treated with other potential plant-based antibacterial agents at 1x MIC value such as artonin E isolated from* Artocarpus communis* [22], gall extract from* Quercus infectoria* [23], and leaf extract from* Urtica dioica* [24].

The slight increment of treated cells diameter as compared to the untreated cells has been observed in previous studies [25–27] by which MRSA isolates have been subjected to 10% NaCl, manuka honey, and oxacillin. The increased cell sizes indicated disruption of cell divisions and slower growth rate in the treated cells [25–27]. Although the cell multiplication was abruptly slowed or stopped, the cellular metabolic processes remained unaffected, hence the increased cell size; that leads to the release of autolytic enzyme under lethal concentrations of inhibitory agents, which in the end resulted in the destruction and deterioration of the treated cells [27]. Additionally, by increasing its size, treated MRSA cells could also reduce their attachable surfaces against antibacterial compounds, hence, tolerating the stress conditions better than normal untreated cells [27]. This survival mechanism corroborated with earlier MBC/MIC ratio value of 32 for ATCC BAA-1688 against MFM501 [1]. Bacterial isolate that showed MBC/MIC ratio of ≥32 was considered tolerant or resistant towards the used anti-infective agent [1].

**Figure 5:**
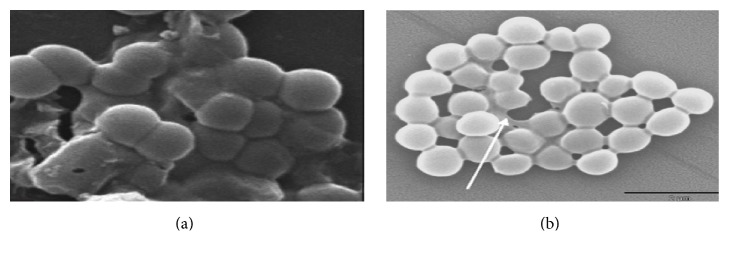
(a) MRSA cells treated with 20 *µ*g/ml of silver nanoparticles after 24 h of incubation (magnification: 37,000x). Image taken from Ansari et al. (2015). (b)* S. aureus* cells treated with 100 *µ*g/ml of vancomycin after 24 h of incubation (no indication of magnification size). Arrow showing a more prevalent extracellular matrix (biofilm). Image taken from Onyango et al. (2013).

Biofilm formation or the production of extracellular matrix by* S. aureus* and/or MRSA isolates has been established as an important virulence factor and survival mechanism for this superbug [28]. In this study, the formations of biofilms were apparent in the treated MRSA cells against MFM501. Although biofilm formations were usually associated with the survival and the indicating resistant bacterial strains, the biofilms produced by BAA-1688 were considered more towards cell deterioration and destruction. As exemplified in Figure 5, the SEM pictographs of treated MRSA cells taken from previous studies [25, 29] exhibited similar biofilm-engulfed cells and cell division disruption as observed in Figure 4 in this study. These comparable biofilm formations were due from the treatment of vancomycin and silver nanoparticles [25, 29]. Based on these earlier studies [25, 29], both compounds deterred* S. aureus*/MRSA pathogenesis by initiating the detachment of biofilm matrix from* S. aureus *cells which eventually inhibited bacterial colonization on the respective surfaces. Hence, it could be postulated that MFM501 may have similar mechanism of action as vancomycin and silver nanoparticles against biofilm-producing* S. aureus*/MRSA isolates.

The results of the acute toxicity test substantiated earlier* in vitro* cytotoxic assay that showed MFM501 was relatively nontoxic against tested three mammalian cell cultures [1]. In addition, according to the GHS acute toxicity scheme, the estimated LD_50_ value for MFM501 was more than 300 mg/kg and less than 2000 mg/kg (Category 4). This classification was for chemicals that were harmful if swallowed while carrying a “Warning” signal word [14]. Although MFM501 was categorized as harmful if swallowed, other common oral antibiotics such as clindamycin, doxorubicin, and clarithromycin shared the same GHS Category 4 [30, 31]. These results showed that MFM501 could be classified with the above-mentioned antibiotics.

An increased AST level in the biochemistry test usually indicated liver injury or myocardial infarction since AST enzyme was found mainly in the liver and heart, although AST could also be detected in kidney, brain, and muscle tissues [32, 33]. However, longer subacute (28 days) and subchronic (30 days) studies have shown that elevated AST level did not cause any visible damage on the liver and heart of treated animals based on its respective histopathological examinations [32, 34]. These observations were substantiated in this study which also displayed no visible damage on the related organs via gross macroscopic evaluation during the shorter period of acute toxicity study (14 days). Vice versa, the lack of any visible changes or damage on the liver and/or heart of treated mice could also be attributed to relatively short duration of exposure to either compound during the acute toxicity test as compared to subacute and subchronic toxicity studies which employed more observational days.

In the mice protection assay, 100% protective ability of linezolid at 25 mg/kg from MRSA infection as described in previous study [35] was corroborated. Currently, only two oral antibiotics against MRSA infections are available, the recently FDA-approved tedizolid (in 2014) and linezolid [36]. In most developing countries, linezolid is the next best antibiotic after vancomycin to combat MRSA infections. Additionally, a previous study [15] showed that the ED_50_ value of linezolid against MRSA infection was at 15.6 mg/kg which was lower than MFM501 that scored ED_50_ value of 87.16 mg/kg. This result showed that MFM501 was not superior to the existing last line of defense against MRSA.

However, there were other published reports [37, 38] on compounds that have higher ED_50_ values than linezolid such as the potential FabI inhibitor, AFN-1252, which has an ED_50_ value of 29.4 mg/kg, and the new oxadiazole compound that displayed an ED_50_ value of 44 mg/kg. On the other hand, higher ED_50_ value for MFM501 was due to its higher MIC values as compared to linezolid. While MIC values for MFM501 were between 15.6 and 31.3 *µ*g/ml against MRSA and MSSA isolates [1], previous studies [37, 38] have shown that MIC value for linezolid against MRSA and MSSA strains was between 0.5 and 2.6 *µ*g/ml.

Nonetheless, since MFM501 could provide moderate 62.5% protection at 125 mg/kg against MRSA infection in a mice model, this experiment also revealed that MFM501 could survive the various barriers of an oral administration route such as the liver metabolism mechanism, degradation by digestive enzymes and acid in the stomach, and interference of absorption by ingested substances in the treated mice [39].

Although less effective than linezolid, these discoveries of potential anti-MRSA agents are medically important since there were numerous reports on the detection of linezolid-resistant* S. aureus *(LRSA) from around the globe [40] Additionally, linezolid too has several adverse side-effects such as reversible myelosuppression, peripheral and optic neuropathy, and lactic acidosis, especially with prolonged use in patients [41, 42].

## 5. Conclusion

Based on the time-kill assay, a concentration-dependent killing mechanism with obvious bacteriostatic effect was observed for MFM501 while SEM analysis suggested that MFM501 may exert its inhibitory activity via cytoplasmic membrane disruption against the two MRSA isolates (ATCC 33591 and ATCC BAA-1688, resp.). Moreover,* in vivo* studies showed that the estimated LD_50_ value for MFM501 was more than 300 mg/kg and less than 2000 mg/kg (Category 4) while exhibiting its efficacy in treating 50% (ED_50_) of the mice population from MRSA infection at 87.16 mg/kg dosage. Additionally, although MFM501 at 300 mg/kg dosage displayed an elevated AST enzyme profile in the biochemistry evaluation, no visible changes or damage on the liver and/or heart of treated mice was observed via macroscopic evaluation. Further in-depth studies involving determination of mechanism of action for MFM501 and subacute/subchronic tests are recommended for MFM501 to be developed as a new clinically active, safe, effective, and orally available anti-MRSA agent.

## Figures and Tables

**Table 1:** Body weight of mice receiving MFM501 at a single dose of 300 mg/kg.

Group	Body weight (g)					% of weight change
	Day 0	Day 3	Day 7	Day 10	Day 14	
Control (untreated)	26.3 ± 1.2	27.2 ± 1.7	27.8 ± 1.4	28.2 ± 1.8	29.5 ± 1.2	11.0 ± 3.2
MFM501 300 mg/kg	28.6 ± 4.3	29.3 ± 3.1	29.5 ± 3.4	30.3 ± 2.5	31.7 ± 3.4	10.0 ± 4.3

Values were expressed as mean ± standard deviation (SD) of five mice. *p* < 0.05 was considered statistically significant difference.

**Table 2:** Hematological analysis of untreated and treated mice with MFM501 at 300 mg/kg.

Hematological analysis	MFM501	
	Untreated	300 mg/kg
WBC (×10^9^/L)	8.5 ± 3.7	7.1 ± 1.7
Lymph (×10^9^/L)	6.6 ± 2.5	5.3 ± 1.2
Mon (×10^9^/L)	0.3 ± 0.2	0.2 ± 0.1
Gran (×10^9^/L)	1.6 ± 1.1	1.5 ± 0.6
Lymph (%)	78.4 ± 4.3	75.8 ± 6.9
Mon (%)	3.2 ± 0.7	3.1 ± 0.8
Gran (%)	18.3 ± 3.8	21.1 ± 6.4
RBC (×10^12^/L)	7.9 ± 0.3	8.4 ± 0.3
HGB (g/dL)	13.6 ± 0.5	13.7 ± 0.6
HCT (%)	39.1 ± 1.4	39.4 ± 2.5
MCV (fL)	49.5 ± 1.9	46.6 ± 2.3
MCH (pg)	17.1 ± 0.8	16.1 ± 0.5
MCHC (g/dL)	34.7 ± 0.2	34.7 ± 0.9
RDW (%)	16.5 ± 0.2	15.5 ± 1.4
PLT (×10^9^/L)	753.6 ± 182.9	777.0 ± 114.4
MPV (fL)	4.5 ± 0.3	4.4 ± 0.2
PWD	16.4 ± 0.2	16.2 ± 0.3
PCT (%)	0.3 ± 0.1	0.3 ± 0.1

Values were expressed as mean ± standard deviation (SD) of five mice. *p* < 0.05 was considered statistically significant difference.

**Table 3:** Biochemistry analysis of untreated and treated mice with MFM501 (300 mg/kg).

Biochemistry analysis	MFM501	
	Untreated	300 mg/kg
Liver profile		
Total protein (g/L)	61.1 ± 25.4	61.26 ± 6.47
Albumin (g/L)	36.4 ± 15.0	33.6 ± 2.6
ALP (U/L)	83.6 ± 41.7	82.8 ± 9.98
ALT (U/L)	43.4 ± 19.3	53.2 ± 8.3
AST (U/L)	84.9 ± 36.7	^*∗*^163 ± 72.01
Renal profile		
Uric acid (*µ*mol/L)	74.2 ± 35.9	78.3 ± 25.47
Lipid profile		
HDL_C (mmol/L)	1.8 ± 0.8	1.7 ± 0.25
Triglycerides (mmol/L)	0.8 ± 0.4	0.8 ± 0.1
Glucose (mmol/L)	10.1 ± 4.2	9.1 ± 0.6

Values were expressed as mean ± standard deviation (SD) of five mice. *p* < 0.05 was considered statistically significant difference. ^*∗*^A significant value change was detected.

**Table 4:** Relative mean organ weight to respective mice body weight and macroscopic examination of mice organs treated/untreated with MFM501 (300 mg/kg).

Organs examined	Relative organ weight (g)		Observable changes	
	Untreated	MFM501 (300 mg/kg)	Untreated	MFM501 (300 mg/kg)
Heart	0.41 ± 0.05	0.36 ± 0.05	None	None
Thymus	0.18 ± 0.03	0.20 ± 0.05	None	None
Lung	0.72 ± 0.11	0.71 ± 0.09	None	None
Liver	4.47 ± 0.65	4.10 ± 0.73	None	None
Left kidney	0.46 ± 0.04	0.44 ± 0.04	None	None
Right kidney	0.44 ± 0.04	0.40 ± 0.04	None	None
Spleen	0.43 ± 0.15	0.54 ± 0.12	None	None
Intestine	11.95 ± 1.28	11.18 ± 0.44	None	None
Left ovary	0.022 ± 0.001	0.027 ± 0.002	None	None
Right ovary	0.021 ± 0.001	0.0028 ± 0.002	None	None
Brain	1.28 ± 0.08	1.33 ± 0.16	None	None

Values were expressed as mean ± standard deviation (SD) of five mice. *p* < 0.05 was considered statistically significant difference.

**Table 5:** Survival rates of control and MRSA-infected mice after being treated with MFM501.

Mice groups	Treated/untreated mice	Total of mice surviving	% survive
Control mice	Untreated—MRSA adjuvant only (−ve control)	0/8	0
	MRSA adjuvant + 25 mg/kg linezolid (+ve control #1)	8/8	100
	MHB + 5% mucin only (+ve control #2)	8/8	100
	Healthy and untreated mice (+ve control #3)	8/8	100

Mice treated with MFM501	125 mg/kg	5/8	62.5
	62.5 mg/kg	3/8	37.5
	31.3 mg/kg	2/8	25
	15.6 mg/kg	2/8	25
